# Effects of Grafting on Morphophysiological and Yield Characteristic of Eggplant (*Solanum melongena* L.) Grafted onto Wild Relative Rootstocks

**DOI:** 10.3390/plants9111583

**Published:** 2020-11-15

**Authors:** Ibrahim Musa, Mohd Y. Rafii, Khairulmazmi Ahmad, Shairul Izan Ramlee, Muhammad Asyraf Md Hatta, Yusuff Oladosu, Isma’ila Muhammad, Samuel Chibuike Chukwu, Nur Nadzirah Mat Sulaiman, Arolu Fatai Ayanda, Jamilu Halidu

**Affiliations:** 1 Institute of Tropical Agriculture and Food Security, Universiti Putra Malaysia, Serdang 43400, Selangor, Malaysia; ibrahimmusa@fukashere.edu.ng (I.M.); oladosuy@upm.edu.my (Y.O.); ismuha@gsu.edu.ng (I.M.); chukwu.samuel@ebsu.edu.ng (S.C.C.); gs50487@student.upm.edu.my (N.N.M.S.); gs53735@student.upm.edu.my (A.F.A.); 2 Department of Agronomy, Faculty of Agriculture, Federal University of Kashere, Gombe 0182, Gombe State, Nigeria; 3 Department of Crop Science, Faculty of Agriculture, Universiti Putra Malaysia, Serdang 43400, Selangor, Malaysia; shairul@upm.edu.my (S.I.R.); gs51813@student.upm.edu.my (J.H.); 4 Department of Plant Protection, Faculty of Agriculture, Universiti Putra Malaysia, Serdang 43400, Selangor, Malaysia; khairulmazmi@upm.edu.my; 5 Department of Agriculture Technology, Faculty of Agriculture, Universiti Putra Malaysia, Serdang 43400, Selangor, Malaysia; m.asyraf@upm.edu.my

**Keywords:** eggplant, splice grafting, cleft grafting, scion-rootstock combinations, *Solanum torvum*

## Abstract

Grafting is regarded as an integral component of sustainable vegetable production. It is important in the management of soil-borne diseases, and reports suggest that grafting with viable rootstocks can enhance crop growth and yield. This research was conducted using splices and cleft grafting techniques to investigate graft compatibility among varieties of high yielding eggplant scion (MCV1, MCV2, CCV1, CCV2, CCV3, NCV, and TCV) grafted onto wild rootstocks (MWR, BWR, and TWR) to study their morphophysiological and yield characteristics. High yielding scions grafted onto wild relative rootstocks were compared with two controls including self-grafted and non-grafted. All the scion had a high rate of germination (≥95%) and remarkable graft success (100%) was recorded in MCV1, MCV2, and TCV using the cleft techniques. Generally, the use of rootstocks resulted in higher total and marketable fruit yield compared to the non-grafted and self-grafted scion plants, respectively. In particular, MWR and TWR rootstock conferred the highest vigour to the scion, resulting in the highest values recorded for total and marketable fruit yield, number of fruits per plant and average fruit weight. A similar result was obtained in fruit length and diameter, where long and wide fruits were observed in scions grafted onto MWR and TWR rootstocks, respectively. Grafting of high yielding eggplant scion onto resistant MWR, BWR and TWR eggplant rootstock was found to be beneficial for eggplant cultivation. The remarkable compatibility and vigour of the rootstock with scion led to the improvement in total and marketable yield of the fruits. As such, it can be concluded that the use of wild relative rootstocks of eggplant species can be a valuable method of improving eggplant production.

## 1. Introduction

Eggplant (*Solanum melongena* L.) is one of the most cultivated vegetables in temperate and tropical regions of the world. It differs in colour, shape and fruit size. The most widely cultivated type is the purple cultivars [1]. The colour depends on content of anthocyanins present in the fruit skin. Anthocyanins are powerful antioxidants group that are located in vacuoles of plant cell belonging to phenolic flavonoids. Eggplant skin extracts have shown high capacity for inhibiting inflammation that leads to atherosclerosis and suppressing metastasis and tumours growth in the blood vessels by developing scavenging free radicals that can damage proteins and lipids [2]. As reported by Yang [3], eggplant was ranked in the top 10 for SOS activity (superoxide scavenging) among 120 vegetable species evaluated based on their antioxidant activity. Additionally, flavonoids isolated from eggplant have shown a great antioxidant activity and their presence in diet is associated with lower risk of heart disease, lung cancer and stroke [2]. 

Despite the high economic importance of this crop, it is susceptible to many pest and diseases particularly insects, nematodes, bacterial wilt, Verticillium and Fusarium wilts [4]. As reported by Moncada et al. [2], more than 78% yield losses were recorded due to soil-borne pests and pathogens such as *Meloidogyne spp.*, Fusarium and Verticillium stroke [2]. For the past few decades, the primary objectives in crop production have been to increase yield and productivity to cater for the increasing world population. As a consequence, limited adoption of crop rotations under intensive cropping systems for vegetable farming in both open field and control environment has built up a negative influence on yield and yield quality as a result of biotic or abiotic factors. These negative effects have led to a consequent increase in use of agricultural input such as pesticides, inorganic fertilizer and water use, which in turn has a detrimental effect on health and environment. The absence of effective chemicals for controlling diseases, coupled with sexual incompatibility between wild and elite cultivars, indicates that a short-term approach to the problem of cultivating susceptible eggplant is the grafting of elite cultivars on rootstocks with multiple pathogen resistance [4]. 

Grafting is a significant integrated approach to the management of biotic and abiotic limitations, fruit yield and quality of solanaceous and cucurbitaceous crops. Management of biotic and abiotic stress conditions is one of the major reasons for the development and use of vegetable grafting technique [2]. This technique has been successfully used in the management of diseases such as *Verticillium, Fusarium*, *Ralstonia*, root-knot nematodes and several soil-borne pathogens [2]. Hence, vegetable grafting onto resistant rootstocks is a tool for the improvement of susceptible high-yielding scion against biotic and abiotic stresses [3]. Despite the comparatively high cost of grafted plants resulting from the higher labour and production input compared to normal (un-grafted) plants, grafting has developed into a specific cultural practice that helps to increased yield and production output and improves economic viability in sustainable vegetable production under both open field and glasshouse conditions [1]. 

Research has shown that positive relationships exist between plants grafted on resistant and vigorous rootstocks, and improvements in production and fruit quality parameters (e.g., phytochemical content, such as phenolic compound [2]. Today, the resistance and fight against soil-borne diseases remain the major objective for applying the grafting technique. It is an effective and fast method for selecting desirable crop characteristics than species hybridization, selection processes or natural screening [5]. Eggplant scions grafted with tomato rootstock have significant increase in fruit yields as a result of increased fruit size and number of fruits in comparison self-rooted plants and self-grafted eggplants [5]. Grafting eggplant onto interspecific eggplant hybrids such as SI×SM hybrid has proved advantageous for eggplant production, as the high vigour and good compatibility of the rootstock with the scion results in improved early and total yield without negative effects on apparent fruit quality or composition [2]. Studies on grafting between *S. melongena* and *S. incanum*, *S. aethiopicum* and *S. habrochaites* germplasm, as well as intraspecific and interspecific eggplant hybrids, have shown promising results for developing new rootstocks for eggplant production [2]. As reported by [2,3,4], grafting can influence yield and fruit quality in eggplant. Generally, grafting increases precocity and plant health, providing more vigorous and stronger root systems compared to un-grafted plants, and the major advantage of this is improved nutrient absorption and water use efficiency. Furthermore, grafted plants provide fruit that is considerably larger than un-grafted plants, while other fruit traits such as soluble solids, skin colour, flesh texture, and shape are negatively influenced by the rootstock despite being under genetic control [2]. Therefore, the objective of this research is to validate the assumption that the use of wild relative rootstocks may be a good approach to enhance eggplant performance by exploiting the advantage of grafting to improve eggplants rooting systems and vigour through the affinity between rootstock and scion.

## 2. Results

### 2.1. Observation of Seedlings at the Nursery Stage and Grafting Success (%)

**Figure 1:**
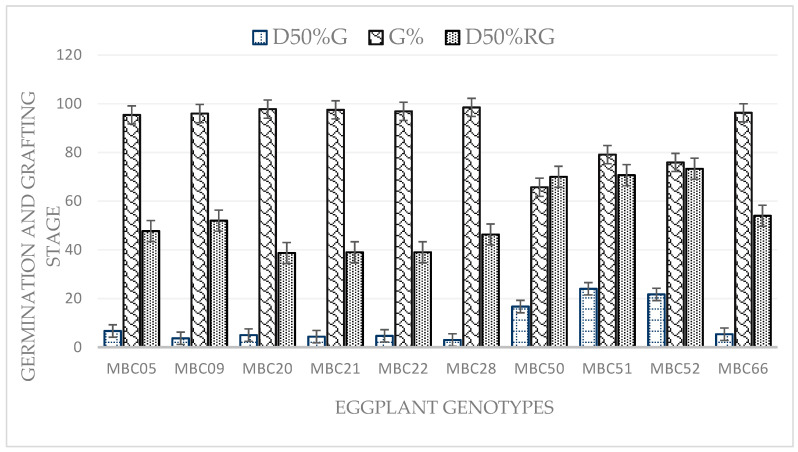
Day to 50% germination (D50%G), germination percentage (G%) and days to 50% reach the grafting (D50%RG) stage, with significance level *p* = 0.05.

**Figure 2:**
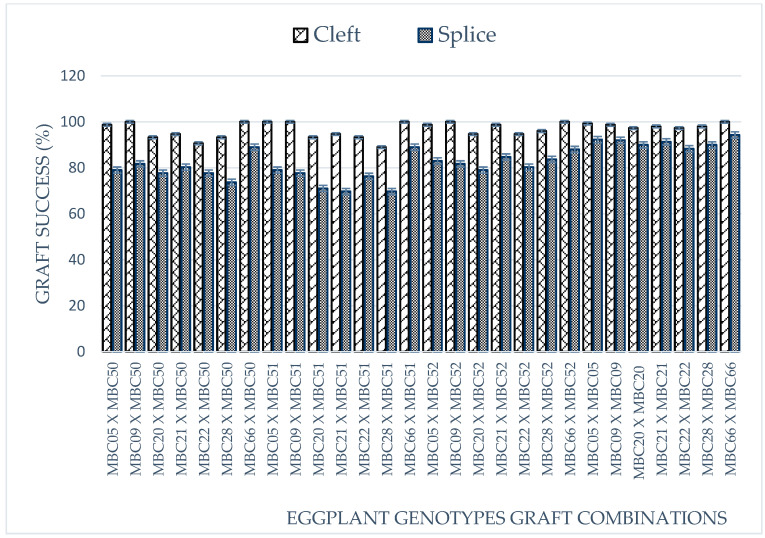
Percentage of graft success (%) with cleft and splice techniques, with significance level *p* = 0.05.

The results indicate that there were highly significant (*p* < 0.01) differences between the different varieties in terms of seed germination, germination percentage (%) and days for the seedling to reached the grafting stage (Figure 1). Among the varieties studied, the earliest germination (3 days) was observed in NCV, followed by MCV2 (3.67 days after sowing), whereas BWR (wild type) germinated later, 24 days after sowing. However, the cultivated genotypes germinated from 3 to 7 days after sowing, while the wild relatives MWR, BWR and TWR germinated between 16 to 24 days after sowing. All the genotypes exhibited a high percentage of germination (>90%) except for the wild relatives (Figure 1). The highest germination percentage (98.47%) was recorded in NCV, followed by CCV1 with 97.83%, whereas a significantly lower (*p* < 0.05) germination percentage (65.83%) was recorded in MWR. Grafting with the cleft technique was relatively more successful with over 89% success in all grafting combinations including self-grafted plants compared to the splice method with over 65% success in grafting combinations (Figure 2). Using the cleft technique, the success recorded among graft combinations ranges between 89% to 100% (Figure 2). The highest graft success was observed in MCV2/MWR, TCV/MWR, MCV1/BWR, MCV2/BWR, TCV/BWR, MCV2/TWR, TCV/TWR and TCV/TCV, whereas NCV/BWR has relatively lower graft success. In the splicing technique, graft success was comparably higher in MCV1/BWR than in TCV/BWR, TCV/TWR, CCV2/TWR, NCV/TWR and MCV1/TWR (Figure 2). The lowest graft success was observed in CCV2/BWR and NCV/BWR, respectively. 

### 2.2. Analysis of Variance for Vegetative, Yield and Yield Component Traits across the Cropping Systems

The combined analysis of variance for vegetative, yield and yield component traits is presented in Table 1. Highly significant differences (*p* ≤ 0.01) were observed among the genotypes for earliness and growth traits. On the other hand, highly significant differences (*p* ≤ 0.01) were observed in the interaction between cropping systems with the genotype for all growth and earliness traits except in stem diameter at 90 days after transplant. Additionally, the results indicated that there was no significant difference among replications for growth and earliness traits except for the number of secondary branches at 45 days after transplant, where a significant difference (*p* ≤ 0.05) was observed. Highly significant differences (*p* ≤ 0.01) were observed among the genotypes, cropping system, and interaction between cropping systems with the genotype (G × Cs) in chlorophyll content, photosynthesis rate, stomatal conductance and transpiration rate. Furthermore, the result showed that there were highly significant differences (*p* ≤ 0.01) among replications for traits such as chlorophyll content and transpiration rate, while significant differences (*p* ≤ 0.05) were recorded in photosynthesis rate. No significant differences were observed in stomatal conductance. Highly significant differences (*p* ≤ 0.01) were observed among the genotypes for yield and yield-related traits. The results showed that a highly significant difference (*p* ≤ 0.01) exists in the interaction between the cropping system and genotype for all yield and yield-related traits. In cropping systems, highly significant differences (*p* ≤ 0.01) were recorded for all yield and yield-related traits except for fruit diameter where a significant difference (*p* ≤ 0.05) was observed. The results showed that there were no significant differences in blocks within each cropping systems for all yield and yield-related traits. 

### 2.3. Analysis of Variance of Interaction between Rootstock and Scion for Vegetative, Yield and Yield Component Across the Cropping Systems

The combined analysis of variance for interaction between rootstock and scion on vegetative, yield and yield component traits is presented in Table 2. Highly significant differences (*p* ≤ 0.01) were observed in cropping systems, rootstock, scion and interaction between rootstock and scion (R × S) on earliness and growth traits in this study except in rootstock where significant differences (*p* ≤ 0.05) was recorded in plant height. No significant difference was observed in rootstock effects on transpiration rate. Additionally, the results implied that there was no significant difference among replications for earliness and growth traits. Highly significant differences (*p* ≤ 0.01) were observed among the cropping system for all yield traits except for fruit diameter which showed no significant difference. For rootstock, highly significant differences (*p* ≤ 0.01) were recorded for all yield traits except yield per plant and yield in ton per hectare, where no significant difference was observed. On the other hand, a highly significant difference (*p* ≤ 0.01) was recorded in the interaction between rootstock and scion for all yield traits except for yield per plant and yield in ton per hectare, where significant interaction (*p* ≤ 0.05) was observed.

### 2.4. Effect of Grafting on Early Flowering and Harvesting across Two Cropping Systems

The mean comparison of vegetative traits is presented in Table 3. The genotypes began flowering at 52 to 85 days from the sowing date. Non-grafted CCV1 produced flower earlier at 52.33 days after sowing (DAS), whereas late flowering was observed in self-grafted MCV2 at 85.17 days after sowing (DAS). For harvesting, the fruits of non-grafted CCV1 were harvested earlier at 63.67 days after sowing (DAS), whereas fruits of self-grafted MCV2 were picked later (95.33 DAS). 

In this study, the number of secondary branches in grafted plants was significantly higher than non-grafted and self-grafted eggplants at 45 and 90 DAT (Table 3). A significantly higher number of branches was observed in TCV grafted onto TWR at 45 and 90 DAT (10.00 and 19.28, respectively), whereas the lowest value for this trait was recorded in non-grafted and self-grafted eggplants. In terms of height, non-grafted NCV (72.80 cm) had relatively taller plants compared to eggplants of NCV grafted onto rootstocks of MWR, BWR and TWR, respectively, at 45 DAT. At 90 DAT, NCV grafted onto TWR (147.00 cm) recorded significantly higher plants compared to non-grafted and self-grafted plants (Table 3). Grafting with wild rootstock recorded significantly higher stem diameter compared to non-grafted and self-grafted eggplants at 45 and 90 DAT (Table 3). The highest stem diameter was observed in NCV grafted onto TWR (14.22 mm at 45 DAT and 21.80 mm at 90 DAT, respectively), whereas the lowest value for stem diameter was observed in non-grafted CCV1 (9.02 mm at 45 DAT) and CCV3 grafted onto MWR (12.28 at 90 DAT). 

### 2.5. Effect of Grafting on Physiological, Yield and Yield Component Traits across Two Cropping System

The physiological traits measured include chlorophyll content, photosynthesis rate, stomatal conductance and transpiration rate. There was no influence of rootstock on chlorophyll content in this study (Table 4). The highest chlorophyll content was recorded in non-grafted MCV2 (6.13 mL/cm^2^) while NCV grafted onto BWR showed the lowest value (3.80 mL/cm^2^). Significant influence of rootstock on photosynthetic rate was observed (Table 4). The highest photosynthetic rate recorded was observed in MCV2 scion grafted onto MWR rootstock (32.18 μmol m^−2^ s^−1^), whereas non-grafted MCV2 recorded the lowest value for this trait (Table 4). For stomatal conductance, CCV3 scion grafted onto MWR rootstock recorded the highest conductance (0.75 mmol m^−2^ s^−1^), whereas the lowest value was observed in non-grafted MCV1 (0.20 mmol m^−2^ s^−1^) (Table 4). There was a remarkable influence of grafting on the transpiration rate, MCV1 grafted onto MWR had the highest transpiration rate (6.03 mL cm d^−1^) among the graft combinations. The lowest transpiration rate was recorded in self-grafted MCV1 (2.75 mL cm d^−1^) (Table 4). 

There was a notable influence of grafting with wild rootstock on fruit length (Table 4). MCV2 scion grafted onto MWR wild rootstock (31.37 cm) recorded significantly higher fruit length than the non-grafted (20.05 cm) and self-grafted (25.02 cm) MCV2. In contrast, self-grafting of MCV2 resulted in a significant increase in fruit length compared to the non-grafted plant (MCV2). On average, eggplants grafting on suitable rootstocks had a positive effect on fruit diameter (Table 4). Commercial MCV2 scion grafted onto BWR wild rootstock recorded comparably higher fruit diameter (99.55 mm) compared to the self-grafted (66.40 mm) and non-grafted (36.35 mm) plants. However, within the same genotypes, self-grafted eggplant had higher fruit diameter compared to their non-grafted counterparts.

Results on fruit yield components indicated that grafting with wild rootstocks significantly increased the average fruit weight (Table 4). Average fruit weight in eggplants from MCV2 scion grafted onto MWR rootstock (195.27 g) was significantly higher than controls, while TCV grafted onto MWR rootstock recorded the lowest average fruit weight (25.22 g).

Significant differences were observed among treatments in the number of fruits per plant (Table 4). TCV scion grafted with TWR rootstocks recorded a higher fruit number (193.80) compared to the non-grafted and self-grafted plants. Across cropping systems, self-grafted TCV eggplants (135.55) outperformed their non-grafted (115.33) counterparts. The average marketable yields in the two cropping systems were significantly higher in grafted plants with the MWR rootstocks compared to the control plant MCV2 (Table 4). 

On the average, grafting of MCV2 onto MWR rootstock recorded a higher marketable fruit yield (5.30 kg) in comparison to self-grafted (4.28 kg) and non-grafted (4.03 kg) MCV2. In contrast, marketable fruit yield was higher in self-grafted eggplant than in their non-grafted counterparts, while the least value for this trait was observed in TCV (Table 4).

## 3. Discussion

Plant growth and development is influenced by physiological processes (e.g., photosynthesis) that depend on environmental factors to proceed optimally [6,7]. Research has shown that grafting is an effective method of increasing yield, disease resistance and quality of several vegetable crops [8,9]. It is believed that rootstocks enhance the yield and quality of fruits. This is attainable through the use of rootstocks that is resistant to soil diseases or pests, tolerant to stress conditions, improves nutrients uptake, and give a high vigour to the scion [5,9]. In this study, the effect of grafting eggplant varieties of MCV1, MCV2, CCV1, CCV2, CCV3, NCV and TCV onto the rootstock of different wild relatives’ eggplants, i.e., MWR, BWR and TWR, were tested. The results of the experiment show that grafting can be used to improve the production of eggplant. The advantages of rootstock grafts such as synchronization and high germination rates of the rootstock and scion, as well as remarkable graft success and stand establishment post-transplant both in field and glasshouse, can adequately compensate for the difficulties encountered in the process of producing good grafted plants. 

It is essential to take note of seed germination when materials of wild species are used as rootstocks. Seeds of some wild Solanum species usually have slow germination, requiring up to 24 days to germinate. Aside from this, research has shown that germination percentage often varies between 15–50% in *S. insanum* L. *S. torvum*, *S. integrifolium*, *S. surattense* Burm., *S. khasianum* C.B. Clarke, *S. sanitwongsei* Craib and in hybrids of *S. melongena* × *S. integrifolium* [10]. *S. torvum*, the most commonly grafted Solanum eggplant relative undergo a longer germination period and often has a low germination percentage [10]. Consequently, this has limited the use of *S. torvum* rootstock due to complexity in achieving speedy and homogeneous germination [11]. In this research, it was observed that soft-seeded natural NCV and MCV2 germinate earlier than the others, at 3–4 days, while their wild relatives, i.e., MWR, BWR and TWR, took longer (17 to 24 days) to germinate. High germination percentage (≥90%) was recorded using seed for MCV1, MCV2, CCV1, CCV2, CCV3, NCV and TCV scions. For MWR, BWR and TWR, the rates recorded (≤80%) were slightly lower, despite both the scion and rootstock being germinated under GA_3_ treatment conditions. From our trial, germinating the seeds of this species in the commercial substrate is attainable; however, despite the optimal conditions, their germination can best be described as irregular and somewhat erratic. In this regard, it is pertinent to know that the wild species *S. incanum* has characteristic low and irregular germination.

Grafting success is dependent on the combination of the grafts and compatibility of the rootstock and scion [11]. Eggplant grafting is mostly performed using the cleft or splice grafting approach [9]. In this research, the cleft and splice grafting techniques were both studied. The result obtained indicated that grafting using the cleft technique was very successful with ≥85% success in all grafting combinations including self-grafted eggplants, compared with the splice method with a success rate of ≥65%. This result shows the cleft technique to be a very efficient method of combining the scion and rootstock of eggplants. The lower success rate recorded using the splice grafting method may be suggestive that the method is not suitable for use in eggplants, but rather for tomato, which is phylogenetically related to eggplant [9]. 

There are several reports of success in the grafting of eggplant varieties using the wild S. torvum which is the phylogenetically most distant of the rootstocks under study [2,3,5]. Using the cleft technique, a 100% survival rate was recorded in plants grafted with TCV scion including self-grafted eggplants and treatments combinations such as MCV1/BWR and MCV2 grafted onto all rootstock. The lower success recorded using splice technique for grafting wild rootstocks may be due to graft union. The interaction between rootstock and scion has been frequently observed in many crops, and in this study, it was noted that rootstock source can have a major influence on eggplant vigour, earliness, yield and fruit quality properties. 

Generally, in grafted plants, the formation of graft union between scion and rootstock is necessary for the translocation of food material from the root system to apical parts of the plant and leads to delayed flowering in grafted plants. In this study, greater earliness was observed in most non-grafted plants. Comparable effect of delayed flowering in grafted plants of eggplant and tomato was reported by Moncada et al. [12] and Ibrahim et al. [10]. The concluding phenomenon is attributable to the stress experienced by these plants following the grafting operation [13]. However, in this study, contrasting results were obtained where grafted plants showed early flowering compared to the non-grafted plants. For example, MCV1 grafted onto MWR produced flower earlier than its corresponding non-grafted MCV1. This phenomenon was related to the absence of graft incompatibility and environmental stress. Gisbert et al. [11], explained that when there is no constraint due to incompatibility and abiotic stress, grafted plants may develop faster, thereby resulting in earliness of the grafted plant. There are reports on earliness in eggplant grafted onto two S. Lycopersicum hybrids [13] and melon plants grafted onto Cucurbita rootstocks [14]. Early harvest may be more important for the farmer to capture higher market prices [9]. Generally, non-grafted plants CCV1 produced fruits faster due to flowering earlier than their grafted counterpart. This may be attributed to the delay resulting from the formation of graft union between scion and rootstock and where necessary to translocate food material from the root system to apical parts of the plant, thereby leading to delayed flowering and fruiting in grafted plants. Additionally, delayed harvesting in the grafted plant may be due to the healing crisis and shock during transplanting. The flowering date is an important aspect of vegetable production as it affects fruit harvesting time, which can, in turn, have a direct bearing on fruit quality [9]. In this study, contrasting results were obtained where some grafted plants bear fruits earlier than the non-grafted plants. For example, MCV1 grafted onto MWR produced fruits earlier than the corresponding non-grafted MCV1. Khah [15] reported a similar observation where grafted tomato fruit earlier than the non-grafted ones.

Grafting with vigorous rootstocks (MWR, BWR and TWR) led to improved growth of scion which results in a higher number of branches compared to controls at 45 and 90 DAT. The effect of rootstock on the mineral content in the aerial portion of the plant may be related to the physical properties of the root system, such as lateral and vertical development, which may lead to improved uptake of water and minerals, thereby resulting in a greater number of branches in grafted plants. These findings are in line with the results reported by [11,15]. 

Plant height, which has been widely regarded as an indicator of plant vigour was highest in plants with MWR, BWR and TWR rootstock at both 45 and 90 days after transplant, while the lowest height was observed in plants with self-grafted, non-grafted and BWR rootstock grafts, thus showing that the vigour of the rootstock is essential in conferring scion vigour. The wild rootstock MWR, BWR and TWR had good root systems which ensured better plant height and vigorous growth through the absorption of an optimal level of water and nutrients. As reported by Gisbert et al. [14], the vigorous root system of the rootstock enhances the ability to absorb water and nutrients compared to the non-grafted plants while serving as a better supplier of endogenous plant hormones. Observations similar to this were reported by Passam et al. [16], showing that the impact of grafting on plant height differ according to the rootstock/eggplant’s varieties combined.

The increase in stem diameter was due to the vigour of the wild rootstock. The effect of rootstock on the level of minerals in the aerial portion of the plant may be related to the physical properties of the root system, including lateral and vertical development which may function in improved uptake of nutrients. The vigorous root system of the rootstock enhanced better growth of scion which resulted in larger stem diameter. Buller et al. [17] found that the stem diameter of Cherokee Purple was larger in plants grafted onto the rootstock Maxifort in a soil infested with verticillium wilt. Similarly, Buller et al. [17] noted that stem diameter was larger in scions of an eggplant variety grafted onto *S. torvum*. In contrast, Leonardi and Giuffrida [18] in their study reported that self-grafted eggplants had similar sized or lower stem diameter compared to eggplant grafted onto different tomato rootstock’s.

Chlorophyll content, which has a direct influence on the photosynthetic efficiency of leaves, is a major index in photosynthetic capacity and may be considered as a source of food and energy [19]. Chlorophyll content was higher in grafted MCV1/TWR under the open field condition, whereas non-grafted had the highest chlorophyll content at the glasshouse conditions. In this study, the lowest value for chlorophyll was recorded in the plants of NCV/BWR under open field cropping system and self-grafted NCV/NCV under the glasshouse environment. Trinchera et al. [19] reported that grafted plants had a relatively higher concentration of chlorophyll pigments compared to controls. Kumar et al. [20] stated that the reduction of water content often leads to increased chlorophyll content. In the incompatible graft combinations, when the synthesis of vascular bundles in the graft union decrease, water and nutrient transport will be inhibited [19]. This may, however, be responsible for higher chlorophyll content at the graft union. Liu et al. [21], in an experiment on tomatoes, found that chlorophyll content was significantly higher in the grafted tomatoes than in the non-grafted ones. 

The photosynthetic rate may be considered as the amount of food manufactured and energy released to other parts of the plant. The rate of photosynthesis was higher in MCV2 grafted onto MBC5 under both cropping systems, whereas the lowest value for this physiological trait was recorded in non-grafted MCV2. A study by Kumar et al. [20] reported that better uptake of nutrients in grafted seedlings leads to improved photosynthesis, which is evident especially when plants are subjected to adverse condition. Stomata conductance, measuring the degree of stomatal opening and serves as an indicator of plant water status, was higher in CCV3 grafted onto MBC5 at both cropping systems, whereas the lowest stomatal conductance was observed in non-grafted MCV1. Higher stomatal conductance in a grafted plants may be due to their vigorous root systems, which are often capable of absorbing water, nutrients and enhancing the efficient accumulation of dry matter compared to non-grafted plants. 

The transpiration rate, considered as the rate of water loss by plant depending on the nature of stomata, was higher in MCV1 grafted onto both MWR and BWR at both cropping systems, respectively, whereas the lowest value for transpiration rate was reported in self-grafted MCV1. The rootstock’s vigorous root system enhances the absorption of water, nutrients and efficient accumulation of dry matter compared to the non-grafted plants. Grafting with wild rootstock can enhance transpiration rate. This claim has been supported by several researches such as report by [19,20,21], who stated that grafting with inter-specific rootstocks (*Cucurbita maxima × C. moschata*) improves transpiration rate in grafted plants.

Fruit quality is vital for fruit marketability, and grafting can have an impact on the traits associated with fruit quality [2]. In our study, it was observed that plants grafted with MWR rootstocks had higher fruit length than the non-grafted plants and self-grafted controls. This can be related to changes in the concentration of growth regulators induced by the eggplant rootstock which leads to increased length of the fruit. Johnson et al. [22] recorded an increase in the fruit lengths of eggplant Epic grafted onto Beaufort, while Bekhradi et al. [23] observed longer fruits in melon plant Charleston Grey grafted onto Ferro rootstocks. In terms of fruit diameter, it was observed that eggplants grafted with TWR rootstocks had higher fruit diameter compared to the non-grafted plants and self-grafted plants treatments, respectively. This was explained to be due to changes in the concentration of growth regulators induced by the wild brinjal rootstock which leads to increased diameter of the fruit. Wide fruits have been reported for eggplant Epic grafted on to Beaufort [22], while cucumber Hady scion grafted onto Ferro rootstocks gave the highest value for this trait [23]. Fruit shape in eggplant has high heritability [1], and rootstocks influence fruit length and fruit diameter, possibly due to changes in the concentration of growth regulators induced by the rootstock.

In this study, average fruit weight was also found to be higher in eggplants grafted with MWR and BWR rootstock. This corresponds with results from other studies that showed positive rootstock–scion interactions on the average fruit weight. Bletsos et al. [24] reported that the fruit weight of grafted plants (Tsakoniki grafted onto *Solanum torvum* Swartz) were observed to be higher than in non-grafted plants in two planting seasons; in this respect, grafting eggplant plants onto wild relative eggplant rootstock resulted in higher average fruit and fruit weight when compared with self-grafted and non-grafted eggplants.

The findings from this study indicated that the use of all wild rootstock significantly increased fruit number per plant in MCV2, CCV3 and TCV. However, MCV1 grafted onto MWR, BWR and TWR gave low fruit number compared to their non-grafted counterparts. This result was supported by Yetisir and Sari [25], who stated that higher number of fruits (2.4 fruits/plant) was obtained when cultivar of Crimson Tide was grafted onto the rootstock of Skopje (*Lagenaria* hybrid), whereas fewer fruits were recorded (1.0 fruits/plant) when the cultivar was grafted onto the rootstock of *Cucurbita moschata* (CMO).

The results from this study show that grafting with resistant and vigorous rootstocks, i.e., MWR, BWR and TWR, can enhance the marketable yield of eggplant plants better than non-grafted and self-grafted control. The increase in marketable yields in grafted treatments combinations in this study has also been documented in other research studies. Khah et al. [13] highlighted that grafting can increase the marketable yield of tomato by up to 54%. The enhanced marketable fruit yield in eggplants from MCV2 and CCV3 grafted onto rootstock MWR, BWR and TWR compared to controls MCV2 and CCV3 under the open field and glasshouse environments, respectively, can be attributed to increase in the production of endogenous hormones leading to improved water and nutrient uptake. Observations similar to this were reported by Gisbert et al. [11] and Khah [13] who explained that higher marketable yield in grafted plants is mainly due to improved water and nutrient uptake by vigorous rootstock.

Generally, the yield improvement in eggplant which occur due to grafting with rootstocks of MWR, BWR and TWR as observed in this study was consistent with previous reports of grafted plant production. Schwarz [26] reported that the use of Maxifort, which is a vigorous rootstock, tends to be highly successful and gives a higher yield of tomato. Louws et al. [5] observed that there was comparatively higher yield increase on grafting eggplant onto wild solanum rootstock than in self-grafted controls. According to Khah et al. [15], a remarkable increase in yield recorded in eggplant grafted onto tomato rootstock was due to increased fruit size and fruit number which was significantly higher than in non-grafted controls and those with eggplant rootstock. Leonardi and Giuffrida [18] and Kumar et al. [20] also reported that increased fruit yield observed in grafted tomato was mainly due to a larger fruit size of the plant.

## 4. Materials and Methods

### 4.1. Planting Materials and Agronomic Practices 

Seven high-yielding eggplant cultivars of *Solanum melongena* were used as scions, among which three were collected from China (CCV1, CCV2 and CCV3), two from Malaysia (MCV1 and MCV2), one was collected from Nigeria (NCV) and Thailand (TCV), respectively. For rootstock, three wild eggplant cultivars of *Solanum torvum* from Malaysia (MWR), Bangladesh (BWR) and Thailand TWR) were used (Table 5). The scions and rootstocks were used to develop 21 interspecific grafted combinations, while the control (seven self-grafted and seven non-grafted scion cultivars) was used as comparison. The 35 grafted, self-grated and non-grafted eggplant seedlings were evaluated under two cropping systems from June to December 2019 for the open field experiment and November 2019 to May 2020 for glasshouse condition. The experiment was laid out in a Randomized Complete Block Design (RCBD) in three replications with a planting distance of 50 cm within plant and 70 cm between the row in each growing system in Field 15, Universiti Putra Malaysia (UPM), Serdang, Selangor, Malaysia, which is geographically located between 3°02’ N latitude and 101°42’ East longitude, at 31 m above sea level altitude. Average minimum and maximum temperature were about 24 °C and 33 °C and 24 °C and 38 °C, respectively, for open field and glasshouse conditions. The mean values of relative humidity were 89 and 78 for the open field and glasshouse, respectively. The mean rainfall during the growing period was 934.7 mm and 743.4 mm, with a monthly mean of 233.7 and 195.9, respectively. The eggplant materials were transplanted to the open field and glasshouse after 15 days of hardening after grafting procedures, with ten plants for each treatment. Agronomic practices and plant maintenance such as fertilizer application, pest and disease management, and weeding were carried out as recommended by the Department of Agriculture, Malaysia (http://jpn.penang.gov.my/index.php/perkhidmatan/teknologi-tanaman/sayur-sayuran/78-terung-sp-3424) for the open field trial, while the Fertigation System Manual [9,10] was used for the glasshouse trial. Plants were examined for insect, pest and disease incidence. Pesticides were regularly applied at the appropriate growth stages of the plant. Daily visit to the study site was conducted for crop maintainance, to observe crop performance and irrigate them when necessary using a rain port sprinkler system. The open field cropping system was fertilized using compound fertilizer NPK 15-15-15+2S and NPK 12-12-17-2+8S+TE following the recommended dose. The first and second doses were applied at two and six weeks after the transplant. The fertigation system in the second study environment (glasshouse) was laid out following the Fertigation System Manual (first edition) recommended by MARDI. Plants were given a daily dosage of copper standard formulation fertilizer comprising of N 14.29, P 1.94, K 7.67, Ca 4.24, Mg 2.06, Fe 0.22, Mn 0.042, B 0.14, Zn 0.0015, Cu 0.0016 and Mo 0.0021, (mmol L^−1^) with electrical conductivity (EC) reading increasing progressively following.

### 4.2. Seed Germination and Grafting Methods

**Figure 3:**
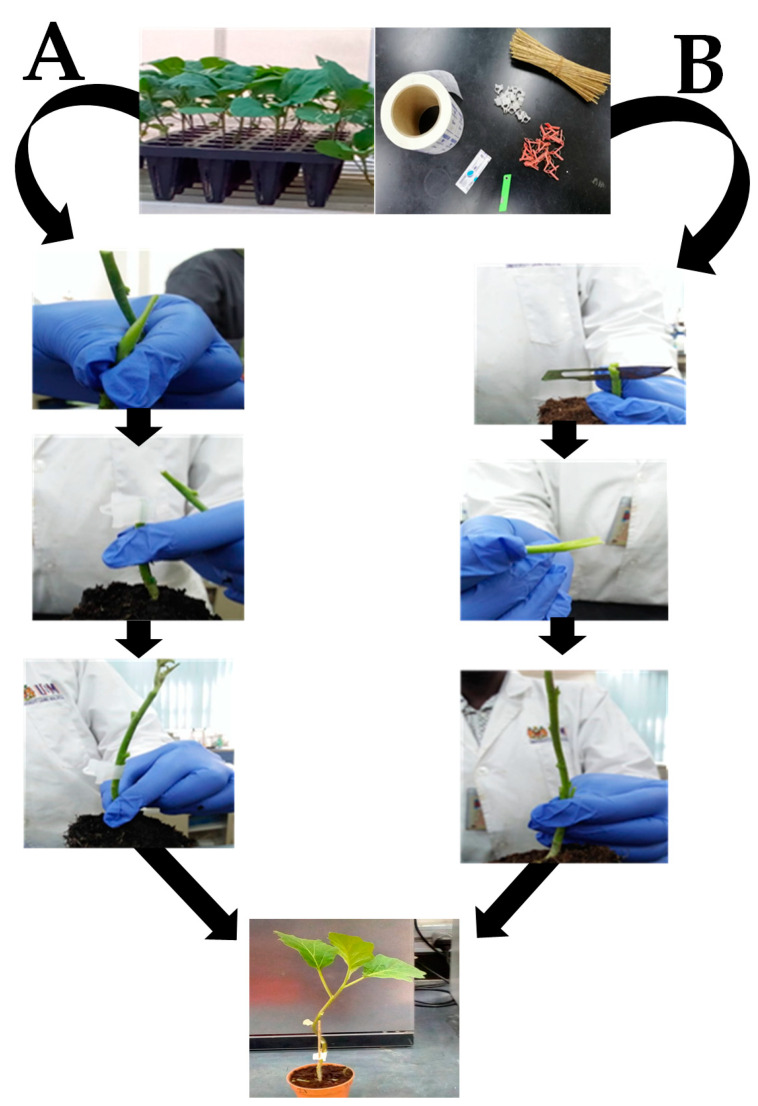
Grafting procedure for splice grafting (**A**) and cleft grafting (**B**) techniques.

The seeds of wild relatives and commercial varieties were soaked in GA_3_ for 24 h before sowing to facilitate early, good and uniform germination. Seeds of rootstock (MWR, BWR and TWR) were sown 2 and 4 weeks before sowing the scion seeds. The scions were sown at three different stages at 2, 4 and 6 weeks. The rootstock seeds were sown in seed trays with 108 holes containing peat moss and later transplanted into 24 celled pro-trays at the two-leaf stage. Two grafting techniques (splices and cleft techniques) were used for this research on 35 days old seedlings (Figure 3), as described by [9]. The experiment was laid out in a Completely Randomized Design (CRD) in three replications. For the splice grafting technique, the rootstock stem was cut at 45° below the cotyledons, a clip was placed over the rootstock stem, and the scion was also cut at 45° above the cotyledons. The scion was placed over the rootstock and the graft was held together using a grafting clip. For the cleft grafting technique, the rootstock stem was cut horizontally below the cotyledons and the top was discarded. The cut was a 0.5-cm-long vertical incision into the centre of the rootstock stem. The scion stem was cut in the same diameter as the rootstock stem diameter at 0.5 cm long wedge shape cut, then the scion stem was inserted into the vertical incision of the rootstock. A plastic clip was placed around the graft union to hold the plant firmly together. 

### 4.3. Data Collection 

From each grafted, self-grafted and non-grafted genotype, five plants were randomly sampled from each replication for data collection. The data on morphological, physiological and yield characteristics were collected after transplanting (Table 6). The measurement of total chlorophyll content was done following Coombs et al. (1987) at the fruiting stages with the formula chlorophyll-a = [(13.19 × A664) − (2.57 × A647)], chlorophyll b = [(22.1 × A647) − (5.26 × A664)], Total chlorophyll = chlorophyll a + chlorophyll b, and Actual chlorophyll = (3.5 × total chlorophyll)/1. The content was measured in actively growing leaf from the third or fourth fully expanded leaves from the tip, and was determined after extraction in 80% (*v*/*v*) acetone/water. Photosynthetic rate, stomatal conductance, and transpiration rate for the 35 treatments were measured at the fruiting stages using an infrared gas analyzer model Li-6400XT (Li-cor Inc., Lincoln, NE, USA). The measurement was conducted on young fully expanded and exposed leaves (third and fourth leaf from the tip) of the tagged plants from each treatment between 9:00 am to 11:00 am when stomata opening was at the optimal point.

### 4.4. Statistical Analysis

The analysis of variance (ANOVA) was carried out on 35 accessions consisting of interspecific grafted (21), self-grafted (7) and non-grafted (7) scions evaluated in two cropping systems, namely, open field and glasshouse, using SAS software version 9.4 (SAS Institute, Inc., Cary, N.C., USA). The accession means comparison was determined using the Least Significant Difference (LSD) at *p* = 0.05 significant level. 

## 5. Conclusions

Grafting technology has the potential to promote the production of eggplant in marginal and fragile agro-ecosystem and has proven to be a rapid alternative tool to confer resistance to bacterial wild disease, promote plant vigour, increase fruit yield and fruit quality in eggplant production. Since grafting gives increased vigour to crops, it will be valuable in sustainable low input horticulture of the future. In Malaysia, where the eggplant production is still carried out mostly through conventional methods and modern production techniques are limited, the grafting technique could help in solving many problems related to eggplant production. Grafting with the cleft technique exhibited high graft success and eggplant scions of MCV1, MCV2 and TCV grafted onto all rootstock displayed good vigour as well as excellent graft compatibility. Overall, the fruit character (fruit length and diameter) recorded was highest in scions of MCV2 and CCV3 grafted on MWR and TWR, respectively. Higher yields were obtained in eggplants with scions of MCV2 and CCV3 grafted onto rootstocks of MWR and TWR respectively, whereas the highest number of fruits were recorded in TCV grafted onto TWR rootstock. The effect of three rootstocks on the agronomic features of the seven eggplant varieties (scions) allow for considerations of the feasibility of this technique on the varieties tested. The grafted plants produced higher yield with better economic value than their self-rooted counterpart. Our results demonstrated that the use of wild relative rootstocks of eggplant species offers a valuable approach for improving eggplant production. 

## Figures and Tables

**Table 1:** Analysis of variance for earliness and growth characters of the grafted, self-grafted and non-grafted eggplants genotypes studied across the cropping systems.

**Source of Variation**	**df**	**D50%F**	**FH**	**NSB45DAT**	**NSB90DAT**	**PH45DAT**	**PH90DAT**
Cropping systems (Cs)	1	262.98 **	123.43 **	993.65 **	3554.70 **	30391 **	93773 **
Blocks within Cs	4	0.86 ^ns^	5.02 ^ns^	0.40 **	0.82 ^ns^	5.5487 ^ns^	9.5181 ^ns^
Genotypes (G)	34	546.39 **	530.70 **	9.72 **	42.72 **	158.50 **	1332.80 **
G × Cs	34	45.03 **	51.68 **	2.93 **	13.81 **	252.88 **	6555.00 **
Error	136	0.40	1.59	0.10	0.82	7.76	8.44
**Source of Variation**	**df**	**SD45DAT**	**SD90DAT**	**PR** (μmol m^−2^ s^−1^)	**SC** (mmol m^−2^ s^−1^)	**TR** (mL cm d^−1^)	**CC** (ml/cm^2^)
Cropping systems (Cs)	1	111.76 **	148.75 **	1000.60 **	0.20 **	34.49 **	44.44 **
Blocks within Cs	4	3.08 *	1.60 ^ns^	0.80 *	0.01 ^ns^	0.07 **	0.20 **
Genotypes (G)	34	10.37 **	28.27 **	49.57 **	0.06 **	4.66 **	3.01 **
G × Cs	34	2.26 **	1.81 ^ns^	1.93 **	0.01 **	0.32 **	0.13 **
Error	136	0.89	2.41	0.31	0.01	0.01	0.01
**Source of Variation**	**df**	**FL** (cm)	**FD** (mm)	**AFW** (g)	**NFPP**	**MFY** (kg)	
Cropping systems (Cs)	1	145.97 **	37.04 *	1433.57 **	8510.96 **	53.56 **	
Blocks within Cs	4	1.29 ^ns^	16.28 ^ns^	67.06 ^ns^	58.51 ^ns^	0.01 ^ns^	
Genotypes (G)	34	257.95 **	2048.90 **	18,502.06 **	130,551.80 **	4.01 **	
G × Cs	34	4.95 **	97.61 **	2548.87 **	661.94 **	4.36 **	
Error	136	1.04	9.62	31.16	34.32	0.01	

* Significant at *p* ≤ 0.05; ** Highly significant at *p* ≤ 0.01; ns, not significant at *p* > 0.05; df, degrees of freedom. D50%F, days to 50% flowering; FH, first harvest; NSB45DAT, number of secondary branches at 45 days after transplanting; NSB90DAT, secondary branches at 90 days after transplanting; PH45DAT, plant height at 45 days after transplanting; PH90DAT, plant height at 90 days after transplanting; SD45DAT, stem diameter at 45 days after transplanting; SD90DAT, stem diameter at 90 days after transplanting; PR, photosynthesis rate; SC, stomata conductance; TR, transpiration rate; CC, chlorophyll content; Fl, fruit length; FD, fruit diameter; AFW, average fruit weight; NFPP, number of fruit per plant; MFY, marketable fruit yield.

**Table 2:** Analysis of variance for interaction between rootstock and scion across cropping systems.

**Source of Variation**	**df**	**D50%F**	**FH**	**NSB45DAT**	**NSB90DAT**	**PH45DAT** **(cm)**	**PH90DAT** **(cm)**
Cropping system (Cs)	1	613.37 **	360.07 **	574.72 **	2407.96 **	18,089.32 **	46,712.82 **
Blocks within Cs	4	0.39 ^ns^	2.60 ^ns^	0.15 ^ns^	0.45 ^ns^	12.56 ^ns^	7.84 ^ns^
Rootstocks (R)	2	3045.10 **	3112.45 **	34.94 **	241.92 **	172.20 *	3256.27 **
Scions (S)	6	152.20 **	169.61 **	7.33 **	15.05 **	196.37 **	727.81 **
R × S	12	297.37 **	280.65 **	9.59 **	34.42 **	161.28 **	1443.39 **
Error	100	5.33	6.69	0.71	3.73	49.61	133.70
**Source of Variation**	**df**	**SD45DAT** (mm)	**SD90DAT** (mm)	**PR** (μmol m^−2^ s^−1^)	**SC** (mmol m^−2^ s^−1^)	**TR** (mL cm d^−1^)	**CC** (ml/cm^2^)
Cropping system (Cs)	1	61.66 **	82.85 **	401.43 **	0.13 **	18.86 **	26.64 **
Block within Cs	4	1.32 ^ns^	2.03 ^ns^	0.46 ^ns^	0.01 ^ns^	0.03 ^ns^	0.13 ^ns^
Rootstocks (R)	2	25.46 **	126.54 **	48.89 **	0.01 **	0.15 ^ns^	13.62 **
Scions (S)	6	5.47 **	29.07 **	6.34 **	0.02 **	0.53 **	3.18 **
R × S	12	10.40 **	20.94 **	26.10 **	0.05 **	2.81 **	1.88 **
Error	100	1.02	2.45	0.32	0.01	0.11	0.05
**Source of Variation**	**df**	**FL** (cm)	**FD** (mm)	**AFW** (g)	**NFPP**	**MFY** (kg)	
Cropping system (Cs)	1	104.38 **	1.46ns	5893.89*	1922.71 **	35.51 **	
Block within Cs	4	1.29 ^ns^	2.80ns	101.93ns	25.89ns	0.00 ^ns^	
Rootstocks (R)	2	2167.44 **	1195.31 **	99,079.51 **	109,510.59 **	8.87 **	
Scions (S)	6	59.67 **	1274.15 **	8753.32 **	4338.70 **	1.37 ^ns^	
R × S	12	44.21 **	3114.52 **	14,525.13 **	4645.01 **	3.44 **	
Error	100	1.82	22.24	618.25	131.35	1.18	

* Significant at *p* ≤ 0.05; ** Highly significant at *p* ≤ 0.01; ns, not significant at *p* > 0.05; SOV, source of variation; df, degrees of freedom. D50%F, days to 50% flowering; FH, first harvest; NSB45DAT, secondary branches at 45 days after transplanting; NSB90DAT, secondary branches at 90 days after transplanting; PH45DAT, plant height at 45 days after transplanting; PH90DAT, plant height at 90 days after transplanting; SD45DAT, stem diameter at 45 days after transplanting; SD90DAT, stem diameter at 90 days after transplanting; PR, photosynthesis rate; SC, stomata conductance; TR, transpiration rate; CC, chlorophyll content; Fl, fruit length; FD, fruit diameter; AFW, average fruit weight; NFPP, number of fruit per plant; MFY, marketable fruit yield.

**Table 3:** Means of growth characters of the grafted, self-grafted and non-grafted eggplant genotypes studied across the cropping systems.

Scion/Rootstock	D50%F	FH	SB45	SB90	PH45 (cm)	PH90 (cm)	SD45 (mm)	SD90 (mm)
MCV1	73.83 ^i^	84.00 ^hi^	5.57 ^p–s^	15.83 ^cd^	56.03 ^k–p^	85.55 ^u^	9.57 ^lm^	13.85 ^h–0^
MCV2	75.50 ^h^	88.33 ^d–f^	5.65 ^o–r^	15.00 ^d–h^	58.85 ^i–l^	105.58 ^g–k^	10.97 ^f–i^	14.17 ^g–n^
CCV1	52.33 ^t^	63.67 ^q^	4.58 ^xy^	9.88 ^op^	53.40 ^pq^	88.88 ^t^	9.02 ^m^	14.40 ^f–m^
CCV2	57.17 ^rs^	68.83 ^no^	4.97 ^u–w^	11.17 ^l–n^	62.12 ^f–h^	96.38 ^n–p^	10.05 ^h–m^	13.05 ^m–o^
CCV3	58.33 ^pq^	70.00 ^l–n^	4.45 ^y^	13.47 ^j^	55.93 ^l–p^	101.05 ^lm^	9.95 ^i–m^	13.43 ^j–o^
NCV	72.50 ^j^	83.17 ^i^	5.70 ^n–q^	15.30 ^d–h^	72.80 ^a^	123.95 ^d^	12.93 ^bc^	18.67 ^b^
TCV	72.33 ^j^	83.67 ^hi^	6.12 ^i–l^	17.00 ^b^	58.70 ^i–l^	89.18 ^st^	10.32 ^g–l^	13.45 ^i–o^
MCV1/MCV1	78.33 ^ef^	89.67 ^cd^	6.22 ^hij^	15.00 ^d–h^	58.62 ^i–l^	89.97 ^rst^	11.17 ^e–g^	14.47 ^i–o^
MCV2/MCV2	85.17 ^a^	95.33 ^a^	5.70 ^n–q^	13.47 ^j^	57.98 ^i–m^	107.58 ^f–i^	11.68 ^d–f^	14.03 ^g–o^
CCV1/CCV1	63.67 ^k^	76.67 ^j^	5.27 ^stu^	14.13 ^h–j^	56.97 ^j–n^	94.98 ^o–p^	10.28 ^g–l^	13.27 ^k–o^
CCV2/CCV2	60.50 ^n^	70.50 ^lm^	4.67 ^w–y^	9.58 ^p^	58.70 ^i–l^	108.08 ^f–h^	9.72 ^lm^	12.57 ^no^
CCV3/CCV3	58.33 ^pq^	70.67 ^l^	4.75 ^v–y^	10.83 ^m–o^	52.17 ^q^	91.85 ^q–t^	9.88 ^i–m^	15.00 ^d–k^
NCV/NCV	76.33 ^g^	85.83 ^g^	5.57 ^p–s^	14.70 ^e–h^	60.12 ^gh^	128.52 ^c^	12.32 ^c–e^	16.37 ^cd^
TCV/TCV	78.17 ^f^	89.00 ^d–f^	8.22 ^d^	16.17 ^bc^	55.10 ^m–q^	92.45 ^q–t^	9.88 ^i–m^	13.55 ^i–o^
MCV1/MWR	76.67 ^g^	87.83 ^f^	5.77 ^l–o^	18.78 ^a^	52.13 ^q^	94.75 ^o–q^	12.63 ^cd^	15.23 ^c–i^
MCV2/MWR	77.83 ^f^	88.83 ^d–f^	6.05 ^i–n^	14.70 ^e–h^	70.10 ^ab^	118.00 ^e^	12.28 ^c–e^	16.93 ^g–o^
CCV1/MWR	56.50 ^s^	66.83 ^p^	5.88 ^k–p^	12.18 ^kl^	59.47 ^g–j^	104.97 ^h–k^	11.03 ^f–h^	13.13 ^l–o^
CCV2/MWR	57.67 ^o^	67.83 ^op^	6.08 ^i–m^	10.65 ^no^	57.78 ^i–n^	104.20 ^j–l^	10.82 ^f–k^	14.88 ^d–j^
CCV3/MWR	59.67 ^o^	71.00 ^l–n^	4.83 ^v–x^	9.37 ^p^	57.55 ^i–n^	102.82 ^kl^	9.58 ^lm^	12.28 ^o^
NCV/MWR	76.83 ^g^	88.17 ^ef^	5.37 ^q–t^	13.50 ^j^	59.27 ^h–j^	135.08 ^b^	13.32 ^a–c^	20.74 ^a^
TCV/MWR	76.83 ^g^	87.83 ^f^	8.58 ^c^	18.70 ^a^	53.71 ^o–q^	106.95 ^f–j^	10.37 ^g–l^	15.63 ^c–f^
MCV1/BWR	78.00 ^f^	90.83 ^bc^	5.67 ^o–q^	14.58 ^i–e^	58.72 ^i–l^	93.20 ^p–r^	11.38 ^e–g^	16.17 ^c–e^
MCV2/BWR	80.33 ^b^	91.50 ^b^	6.58 ^f–h^	15.55 ^c–e^	66.82 ^cd^	122.00 ^d^	12.35 ^c–e^	15.98 ^c–g^
CCV1/BWR	61.33 ^m^	71.33 ^l^	5.95 ^j–o^	13.58 ^ij^	62.60 ^e–g^	108.80 ^fg^	12.32 ^c–e^	16.03 ^c–f^
CCV2/BWR	59.83 ^no^	70.67 ^l^	5.75 ^m–p^	9.87 ^op^	59.60 ^g–j^	93.95 ^o–q^	10.90 ^i–m^	14.45 ^c–h^
CCV3/BWR	62.50 ^l^	73.50 ^k^	4.97 ^u–w^	11.53 ^k–n^	53.47 ^pq^	94.17 ^o–q^	10.85 ^f–j^	12.88 ^m–o^
NCV/BWR	74.50 ^i^	84.83 ^gh^	6.62 ^f–h^	14.20 ^g–j^	65.38 ^de^	135.93 ^b^	13.82 ^a^	18.35 ^b^
TCV/BWR	76.67 ^g^	87.83 ^f^	9.03 ^b^	15.22 ^c–g^	53.57 ^o–q^	96.97 ^o^	11.02 ^f–h^	14.97 ^d–k^
MCV1/TWR	79.50 ^cd^	89.66 ^cd^	6.98 ^e^	16.25 ^bc^	56.70 ^j–o^	91.85 ^q–t^	9.88 ^i–m^	13.93 ^g–o^
MCV2/TWR	79.00 ^de^	88.33 ^d–f^	6.77 ^ef^	14.42 ^f–j^	65.15 ^d–f^	115.33 ^e^	12.43 ^c–e^	16.35 ^cd^
CCV1/TWR	58.33 ^pq^	69.17 ^m–o^	5.30 ^r–u^	10.87 ^m–o^	54.73 ^n–q^	104.60 ^i–k^	10.37 ^g–l^	12.75 ^m–o^
CCV2/TWR	62.17 ^l^	72.83 ^k^	5.05 ^t–v^	11.70 ^k–m^	55.00 ^m–q^	104.28 ^i–l^	9.73 ^k–m^	15.18 ^c–j^
CCV3/TWR	58.83 ^p^	70.83 ^l^	4.83 ^v–x^	11.33 ^k–n^	55.35 ^m–p^	99.10 ^mn^	10.57 ^g–l^	15.58 ^c–g^
NCV/TWR	77.83 ^f^	89.00 ^d–f^	6.25 ^h–j^	14.62 ^e–i^	69.52 ^bc^	147.00 ^a^	14.22 ^a^	21.80 ^a^
TCV/TWR	79.83 ^bc^	89.17 ^d–f^	10.0 ^a^	19.28 ^a^	59.12 ^k^	109.52 ^f^	11.82 ^d–f^	15.48 ^c–h^
Mean	69.52	80.48	6.01	13.75	58.96	105.35	11.13	15.11
SEM	0.68	0.68	0.18	0.36	1.02	1.92	0.12	0.19
LSD (*p* = 0.05)	0.72	1.44	0.35	1.04	3.18	3.32	1.08	1.77

SEM, standard error of mean; LSD, least significant difference; D50%F, days to 50% flowering; FH, first harvest; SB, secondary branches; PH45DAT, plant height at 45 days after transplanting; PH90DAT, plant height at 90 days after transplanting; SD45DAT, stem diameter at 45; SD90DAT, stem diameter at 90. Means with the same letter in the same column are not significantly different at *p* = 0.05 with LSD test.

**Table 4:** Means of physiological, yield and yield component traits of the grafted, self-grafted and non-grafted eggplants genotypes studied across the cropping systems.

Scion/Rootstock	CC	P R	S C	T R	FL (cm)	FD (mm)	AFW (g)	NFPP	MFY (kg)
MCV1	4.72 ^i^	22.10 ^l^	0.20 ^k^	3.43 ^no^	18.9 ^j^	32.6 ^pq^	90.2 ^ij^	54.8 ^l–n^	2.5 ^tu^
MCV2	6.13 ^a^	18.63 ^m^	0.43 ^ef^	3.37 ^no^	20.1 ^h–j^	34.1 ^n–g^	142.6 ^e^	53.8 ^k–m^	4.0 ^fg^
CCV1	4.02 ^p^	23.32 ^j^	0.30 ^j^	3.02 ^q^	15.9 ^kl^	33.3 ^o–q^	95.7 ^i^	51.3 ^m–o^	2.6 ^st^
CCV2	3.98 ^p^	25.35 ^h^	0.33 ^i^	3.37	21.2 ^g^	36.4 ^m–o^	90.5 ^ij^	54.1 ^l–n^	2.3 ^v^
CCV3	4.45 ^k^	25.32 ^h^	0.35 ^hi^	3.30 ^op^	15.7 ^l^	72.2 ^c^	154.1 ^d^	46.0 ^op^	2.9 ^r^
NCV	3.93 ^pq^	25.37 ^h^	0.28 ^j^	3.32 ^n–p^	11.3 ^op^	27.3 ^r^	33.9 ^k–m^	128.2 ^f^	3.2 ^n^
TCV	4.37 ^kl^	24.02 ^i^	0.43 ^ef^	3.48 ^m^	6.53 ^r^	19.7 ^s^	27.8 ^m–p^	115.3 ^g^	2.1 ^w^
MCV1/MCV1	4.37 ^l^	23.32 ^j^	0.35 ^hi^	2.75 ^r^	22.6 ^f^	39.4 ^j–m^	114.4 ^g^	50.2 ^no^	2.8 ^r^
MCV2/MCV2	5.57 ^d^	24.40 ^j^	0.45 ^de^	3.63 ^l^	25.0 ^de^	38.1 ^k–m^	116.1 ^g^	62.7 ^h–j^	4.3 ^e^
CCV1/CCV1	4.82 ^h^	22.12 ^l^	0.30 ^j^	3.22 ^p^	10.5 ^pq^	37.0 ^l–n^	95.0 ^i^	66.6 ^h^	3.8 ^ij^
CCV2/CCV2	4.55 ^j^	22.53 ^kl^	0.35 ^hi^	3.73 ^kl^	25.5 ^de^	30.8 ^q^	85.5 ^j^	55.0 ^l–n^	2.2 ^v^
CCV3/CCV3	4.82 ^h^	22.82 ^jk^	0.42 ^fg^	3.80 ^k^	17.0 ^k^	66.4 ^d^	178.2 ^c^	41.9 ^p^	2.9 ^q^
NCV/NCV	3.85 ^rs^	26.80 ^g^	0.45 ^de^	4.17 ^ij^	13.8 ^m^	52.6 ^f^	25.7 ^op^	177.3 ^b^	3.5 ^lm^
TCV/TCV	4.45 ^k^	25.25 ^h^	0.40 ^g^	3.80 ^k^	8.68 ^q^	27.4 ^r^	28.6 ^m–p^	135.6 ^e^	2.5 ^tu^
MCV1/MWR	6.05^b^	29.35 ^c^	0.50 ^c^	6.03 ^a^	29.4^b^	43.4 ^hi^	134.2 ^f^	54.4 ^l–n^	3.8 ^i^
MCV2/MWR	5.73 ^c^	32.18 ^a^	0.55_b_	5.03 ^e^	31.4 ^a^	47.9 ^g^	195.3 ^a^	52.2 ^m–o^	5.3 ^a^
CCV1/MWR	5.23 ^g^	25.68 ^h^	0.55^b^	4.90 ^f^	25.5 ^de^	38.4 ^k–m^	103.2 ^h^	65.6 ^hi^	4.0 ^fg^
CCV2/MWR	4.18 ^mn^	27.97 ^de^	0.50 ^c^	5.20 ^d^	27.3 ^c^	36.0 ^m–p^	116.1 ^g^	55.1 ^k–n^	3.0 ^pq^
CCV3/MWR	4.22 ^m^	28.43 ^d^	0.75 ^a^	5.80 ^b^	20.4 ^g–i^	90.3^b^	186.7^b^	46.0 ^op^	3.5 ^lm^
NCV/MWR	5.58 ^J^	31.37 ^b^	0.55 ^b^	5.47 ^c^	13.9 ^m^	45.8 ^gh^	38.9 ^k^	130.8 ^ef^	3.8 ^hi^
TCV/MWR	4.57 ^j^	29.41 ^c^	0.55^b^	5.02 ^e^	11.3 ^op^	39.4 ^j–m^	25.2 ^p^	157.6 ^c^	2.6 ^s^
MCV1/BWR	5.37 ^f^	27.95 ^de^	0.43 ^ef^	5.40 ^c^	25.8 ^d^	45.9 ^gh^	136.5 ^ef^	52.2 ^m–o^	3.6 ^jkl^
MCV2/BWR	5.15 ^g^	31.02 ^b^	0.50 ^c^	4.55 ^h^	30.0 ^b^	40.7 ^i–k^	139.9 ^ef^	59.9 ^i–l^	4.9 ^c^
CCV1/BWR	5.23 ^m^	25.78 ^h^	0.55^b^	4.60 ^h^	23.0 ^f^	42.9 ^h–j^	115.9 ^h^	58.9 ^i–l^	3.8 ^hi^
CCV2/BWR	5.17 ^g^	26.80 ^g^	0.50 ^c^	5.07 ^e^	25.8 ^d^	40.1 ^i–l^	103.3 ^h^	61.8 ^h–k^	3.2 ^n^
CCV3/BWR	4.07^o^	28.10 ^de^	0.55^b^	5.47 ^c^	21.1 ^gh^	33.2 ^o–q^	192.3 ^ab^	49.3 ^no^	3.7 ^ij^
NCV/BWR	3.80 ^h^	27.80 ^d–f^	0.55^b^	5.35 ^c^	13.8 ^m^	58.7 ^e^	31.9 ^l–o^	181.4 ^b^	4.6 ^d^
TCV/BWR	4.33 ^l^	27.62 ^ef^	0.50 ^c^	4.57 ^h^	10.9 ^p^	37.1 ^l–n^	33.4 ^k–n^	148.5 ^d^	3.2 ^n^
MCV1/TWR	6.07^b^	27.53 ^ef^	0.45 ^de^	4.63 ^h^	24.7 ^de^	40.7 ^i–k^	136.8 ^ef^	48.9 ^no^	3.1 ^op^
MCV2/TWR	5.50 ^e^	27.58 ^ef^	0.40 ^g^	4.27i	27.2 ^c^	42.5 ^h–j^	172.6 ^c^	54.7 ^l–n^	5.1^b^
CCV1/TWR	4.82 ^h^	24.55 ^i^	0.37 ^h^	4.08 ^k^	24.5 ^e^	39.4 ^j–m^	117.6 ^g^	57.5 ^j–m^	3.7 ^ij^
CCV2/TWR	4.20 ^m^	25.30 ^h^	0.40 ^g^	5.83^b^	22.5 ^f^	28.7 ^r^	86.5 ^j^	57.5 ^j–m^	2.4 ^u^
CCV3/TWR	4.13 ^no^	27.53 ^ef^	0.50 ^c^	4.80 ^fg^	21.2 ^g^	99.5 ^a^	192.8 ^ab^	49.2 ^no^	4.1 ^f^
NCV/TWR	4.13 ^no^	27.17 ^fg^	0.47 ^d^	4.67 ^gh^	12.9 ^mn^	57.6 ^e^	36.2 ^kl^	144.8 ^d^	3.9 ^gh^
TCV/TWR	3.88 ^qr^	27.12 ^fg^	0.45 ^de^	4.27 ^i^	12.2 ^op^	34.4 ^n–p^	27.4 ^n–p^	193.8 ^a^	3.7 ^ij^
Mean	4.68	26.22	0.44	4.35	19.91	45.14	102.89	82.09	3.44
SEM	0.06	0.25	0.01	0.07	0.46	1.30	4.06	3.31	0.09
LSD (*p* = 0.05)	0.08	0.64	0.02	0.13	1.17	3.54	6.37	6.69	0.11

SEM, standard error of mean; LSD, least significant difference; CC, chlorophyll content (mL/cm^2^); PR, photosynthetic rate (μmolm^−2^ s^−1^); SC, stomata conductance (mmolm^−2^ s^−1^); TR, transpiration rate (mL cm d^−1^); FL, fruit length; FD, fruit diameter; AFW, average fruit weight; NFPP, number of fruit number; MFY, marketable fruit yield. Means with the same letter in the same column are not significantly different at *p* = 0.05 with LSD test.

**Table 5:** Accessions (*Solanum melongena*) and species used for this study.

No	Code	Original Code	Type of Material	Fruit Type	Origin
	**Scion**				
1	MCV1	Green world white eggplant 330	Commercial variety	Long	Malaysia
2	MCV2	Green world purple dream eggplant 302	Commercial variety	Long	Malaysia
3	CCV1	China vegetable seed technology 62146129	Commercial variety	Long	China
4	CCV2	China vegetable seed technology 62119631	Commercial variety	Long	China
5	CCV3	China vegetable seed technology 62119631	Commercial variety	Round	China
6	NCV	Yalo garden egg eggplant	Commercial variety	Round	Nigeria
7	TCV	Round eggplant (Chao-phaya)	Commercial variety	Round	Thailand
	**Rootstock**				
8	MWR	MFS eggplant	Wild relatives	Round	Malaysia
9	BWR	Plate brush eggplant	Wild relatives	Round	Bangladesh
10	TWR	Pea eggplant	Wild relatives	Round	Thailand

**Table 6:** Data and description in the nursery, and morphological traits.

Parameters	Denotation	Data Collection
Days for 50% seeds germination	D50%SG	Daily visual observations were recorded until the reaching of 50% of emerged seedlings
Germination %	G%	The observation was recorded at every day by visual observation until all germinated
Days taken to 50% of grafting stage plants	G50%RGS	Daily visual observations were recorded until 50% of plants reached grafting stage
Graft success (%)	GS	A graft success was recorded at 15 days after grafting based on wilting of the grafts at healing region.
Days taken for 50% flowering	D50%F	Number of days from sowing to 50% flowering.
First harvest	FH	Days to first fruit picking was measured by counting the number of days after transplanting to the day of the first picking at the marketable stage.
Plant height (cm)	PH	Plant height 45 and 90 days after transplanting.
Number of secondary branches	NSB	Numbers of secondary branches 45 and 90 days after transplanting.
Stem diameter	SD	The diameter of the stem was measured 5 cm above the ground level at 45 and 90 days after transplanting with an electronic digital caliper.
Fruit length (cm)	FL	The length of one matured fruit per plant from calyx to the apex of the fruit
Fruit diameter (cm)	FD	The diameter of one matured fruit (0.3 cm below the calyx) using venire calipers
Fruit weight (g)	FW	The weight of one matured fruit per plant.
Number of fruits per plant	NFPP	Total number of fruits from the first harvest until 90 days after transplanting
Average fruit weight (g)	AFW	By weighing of individual fruits.
Marketable fruit yield (Kg)	MFY	Total weight of fruit from the first harvest until 90 days after transplanting
